# Efficacy of Venlafaxine and Deep Brain Stimulation Against the Effects of Hippocampal Lesion with Ibotenic Acid in Animals Exposed to the Chronic Mild Stress Model of Depression

**DOI:** 10.2174/011570159X391083250716080839

**Published:** 2025-07-28

**Authors:** Ewa Litwa, Aleksandra Bartosz-Nowakowska, Bartosz Bobula, Piotr Gruca, Magdalena Lason, Dominika Biała, Krzysztof Tokarski, Grzegorz Hess, Mariusz Papp

**Affiliations:** 1 Department of Pharmacology, Maj Institute of Pharmacology, Polish Academy of Sciences, Kraków, Poland;; 2 Department of Physiology, Maj Institute of Pharmacology, Polish Academy of Sciences, Kraków, Poland;; 3 Department of Neurophysiology and Chronobiology, Faculty of Biology, Institute of Zoology and Biomedical Research, Jagiellonian University, Kraków, Poland

**Keywords:** Treatment-resistant depression, neuromodulation, CMS animal model of depression, ibotenic lesion, vHPC and mPFC, DBS and venlafaxine, behaviour and electrophysiology

## Abstract

**Introduction:**

Dysfunction of the pathway between the ventral hippocampus (vHPC) and medial prefrontal cortex (mPFC) may be responsible for the weaker or lack of efficacy of antidepressant drugs in patients suffering from treatment-resistant depression. This study aims to evaluate the behavioural effects of vHPC lesion with ibotenic acid (IBO) in animals subjected to the chronic mild stress (CMS) procedure and treated with either chronic venlafaxine or acute deep brain stimulation (DBS) in the mPFC. In addition, electrophysiological studies are expected to reveal neuromodulatory effects on the function and plasticity of mPFC neurons in response to stress, lesion, and deep brain stimulation (DBS).

**Methods:**

Wistar Han rats were exposed to the chronic mild stress model of depression and IBO lesion in vHPC. The effects of both procedures were evaluated in a series of behavioural tests (sucrose test, elevated plus maze, novel object recognition, and social interaction) and in electrophysiological recordings (field potential recording and LTP induction).

**Results:**

The CMS procedure caused a decrease in sucrose consumption, deficits in cognitive function and social interaction, and increased anxiety. The lesion in vHPC with IBO resulted in similar behavioral changes. Repeated (5 weeks) administration of venlafaxine (10 mg/kg, IP) reversed these deficits in stressed animals but was only partially effective in reversing the effects of IBO lesion in vHPC. In contrast, the neuromodulation strategy with DBS of the mPFC produced a robust reversal of all behavioural changes observed in both stressed and lesioned rats. The CMS did not affect the amplitude of field potentials in mPFC slices, but the induction of Long-Term Potentiation was impaired in these animals. The IBO lesion significantly reduced the amplitude of Field potentials as compared to unstressed rats. Both repeated venlafaxine and acute DBS normalized these effects of the IBO lesion.

**Discussion:**

Observed effects were fully normalized by DBS in mPFC but not by venlafaxine, which only partially reversed the IBO lesion-induced effects. The weaker sensitivity of vHPC-lesioned animals to the therapeutic action of venlafaxine provides further evidence that insufficient transmission from the vHPC to the mPFC could be responsible for antidepressant non-response.

**Conclusion:**

These data support the hypothesis that resistance to antidepressant treatment may result from the inability of antidepressants to fully activate the impaired vHPC-PFC pathway, which could be overcome by the neuromodulatory properties of deep brain stimulation.

## 1 INTRODUCTION

Major depressive disorder (depression) is a common mental disorder that causes a long-term impairment of almost all areas of life and is a major cause of ill health and economic burden, reflecting the high proportion of people with comorbid psychiatric and other health disorders [1, 2]. Treating individuals with depression still presents a significant problem, as antidepressant drugs do not always elicit a full therapeutic response. At least one-third of patients fail to respond to pharmacotherapy and, therefore, are referred to as having treatment-resistant depression (TRD) [3-5]. However, alternative therapies, such as new rapid-acting drugs (*e.g*., ketamine or psilocybin) or deep brain stimulation (DBS), may be effective in treating TRD patients [6, 7] and have shown activity in preclinical studies [8-11].

A review of potential animal models for TRD identified the Wistar-Kyoto (WKY) rats as a prime contender, which have long been considered to be resistant to antidepressant drug treatment [12]. Therefore, in a series of previous studies and this study implemented the chronic mild stress (CMS) procedure in WKY rats and found that they failed to recover following chronic treatment with different antidepressant drugs. Nevertheless, they did show recovery on a battery of behavioural tests following acute and subacute ketamine treatment or acute DBS of mPFC [13-16]. Hence, CMS in the WKY rats appears to provide a valid model of TRD [17].

The therapeutic efficacy of chronic treatment with antidepressant drugs in the CMS model has been shown to involve not only a reversal of stress-induced neurotoxic damage to both the ventral hippocampus (vHPC) and medial prefrontal cortex (mPFC) [18, 19] but also the functional connectivity between these two structures [20, 21]. Taking this into account, our previous studies have shown that DBS of the mPFC, as well as optogenetic stimulation of the vHPC-PFC pathway can normalize anhedonia, anxiety-like behaviour, and cognitive impairment in rats exposed to the CMS procedure. These results led to a hypothesis that a dysfunction of the pathway between vHPC and PFC may be responsible for the weaker effect of antidepressant drugs or even drug resistance in this model [15, 22]. All these suggest that in antidepressant-responsive Wistar Han rats, a permanent destruction of neurons in the vHPC should lead to effects similar to those caused by the CMS procedure, and these effects should be sensitive to DBS but not to antidepressant pharmacotherapy [23-26].

Hence, this study aimed to evaluate the effects of vHPC lesion with the ibotenic acid (IBO) in animals exposed to the CMS procedure and treated with either chronic venlafaxine or acute DBS in mPFC. It was anticipated that a lesion of the vHPC in Wistar Han rats should induce behavioural effects observed in WKY controls and provide information on a possible mechanism for the resistance to antidepressants. Moreover, electrophysiological studies will help to understand the function and plasticity of mPFC neurons in response to stress, lesions, and DBS stimulation. The advantage of IBO over other neurotoxins is that it has less general toxicity and weaker epileptogenic properties, which makes IBO a valuable tool for producing discrete lesions within a studied structure [27-29].

## 2 MATERIALS AND METHODS

### 2.1 Study Design

The CMS procedure begins with a weekly adaptations to consumption of 1% sucrose solution (Chronic Mild stress (CMS) procedure, Fig. **1**). Once stabilisation of the consumption is achieved (usually after 6 weeks), animals are divided into non-stressed (CON) and to-be-stressed (STR) groups, based on their intakes in the final adaptation test. The criterion is comparable to sucrose solution intakes (usually 12-15 g) in the last two tests. Approximately 30% of the animals do not meet the stabilisation criterion and are excluded from the experiment. CON remains undisturbed, except for weekly sucrose consumption tests, stereotaxic procedures, and standard husbandry, which were applied to both groups: CON and STR (see below). Then, the stress procedure is started and after first 2 weeks of initial stress, animals are divided into treatment groups, again this is based on their intakes in final test (according to the criterion of a decrease up to 40% of the pre-stress intakes), detailed description of the procedure: Papp M. and Willner P., 2023 [30]. Matched groups received an IBO lesion or 0.9% NaCl (SHAM). Animals for the DBS experiment were implanted with DBS electrodes during the same surgery. Two weeks later, the lesioned and SHAM animals were divided into treatment groups, *i.e*., the venlafaxine (VEN) or DBS groups. The number of animals in each experimental group is n=8. Timelines are shown in Fig. (**1**).

#### 2.1.1 Experiment 1: vHPC Lesion with IBO in CON and STR Rats Treated with Venlafaxine

VEN (10 mg/kg/day, IP) or Sal (1 ml/kg/day, IP) was administered to CON and STR animals for 5 weeks, during this time weekly sucrose tests were conducted. Then, all animals were exposed to the Elevated Plus Maze (EPM), Novel Object Recognition (NOR), and Social Interaction (SI) tests. Twenty-four hours after the final behavioural test, the animals were sacrificed and their brains were removed to verify the efficacy of the IBO lesion (Fig. **1**, Experiment 1)—total number of animals in experiment: n=64.

#### 2.1.2 Experiment 2: vHPC Lesion with IBO in CON and STR Rats Received DBS in mPFC

Two 2-hour DBS sessions were conducted, one on the previous evening and one on the morning before the 3rd sucrose intake test. Then, all animals were exposed to the EPM, NOR, and SI tests. Twenty-four hours after the final behavioural test, the animals were sacrificed and their brains were removed to verify the location of the DBS electrode tips and the efficacy of the IBO lesion (Fig. **1**, Experiment 2)—total number of animals in experiment: n=64.

#### 2.1.3 Experiment 3: Electrophysiological Analysis in CON and STR Rats Treated with VEN or DBS Following IBO Lesion of vHPC

Separate groups of CON and STR animals received IBO lesion in vHPC, VEN administration, or DBS in mPFC as described in Experiment 1 and Experiment 2 above. Twenty-four hours after the final dose of VEN or 30 minutes after the final DBS session, the animals were sacrificed, and their brains were removed to perform electrophysiological recordings on brain slices (see below).

### 2.2 Animals

Male Wistar Han rats (5 weeks old, approximately 100 g at baseline and 12 weeks old, approximately 330 g before CMS initiation, obtained from Charles River, Germany) were brought to the laboratory one month before the start of the experiment. Except for the first 10 days after arrival, when the animals were housed in groups of 8, they were housed individually with free access to food and water. They were maintained on a 12-hour light/dark cycle (lights on at 08:00) under constant temperature (22 ± 2°C) and humidity (45 ± 5%). All procedures used conformed to the rules and principles of EEC Directive 86/609 and were approved by the 2^nd^ Local Institutional Animal Care and Use Committee at the Maj Institute of Pharmacology, Polish Academy of Sciences, Krakow, Poland (Approval no 314/2021).

### 2.3 Chronic Mild Stress (CMS) Procedure

The CMS procedure was conducted as previously described [18]. Animals were trained to consume 1% sucrose solution. During the weekly 1-hour sucrose consumption test, which was preceded by 14 hours of food and water deprivation, the bottles were weighed before and after the test. Every week the stress regime consisted of: 2 periods of food or water deprivation, 2 periods of 45° cage tilt, 2 periods of intermittent lighting (light on and off every 2 hours), 2 periods of a soiled cage (250 ml water in sawdust bedding), 1 period of grouped housing, 2 periods of low-intensity stroboscopic lighting (150 flashes/min) and 3 periods of no stress. The duration of all stressors was 10 hours, and they were applied individually and continuously, day and night. Control animals were housed in separate rooms and were deprived of food and water for 14 hours only before the sucrose test.

### 2.4 Lesion Procedure

Animals were anesthetized with pentobarbital (60 mg/kg, IP) and placed in a stereotaxic apparatus (Stoelting Co., Wood Dale, IL, USA). Bilateral infusion of IBO into the vHPC was made using an infusion pump (0.5 μg/side, 0.1 μL/min; total volume 0.5 μL) to vHPC (anteroposterior (AP) –5.3 mm, lateral (L) –5.4/+5.4 mm, dorsoventral (DV) –7.4 mm from Bregma) according to the atlas of Paxinos and Watson (2013). Sham animals underwent the same procedure, but they received saline.

### 2.5 Deep Brain Stimulation

#### 2.5.1 Electrode Implantation

A monopolar stainless-steel stimulating electrode (model C315G-MS303/2, Plastics One Inc., Roanoke, VA, USA) consisted of two wires; one wire (250 μm in diameter, 0.75 mm of exposed surface) was attached to the guide cannula and extended 1.4 mm below it and served as cathode; the second wire had an epidural screw attached to the skull and was used as anode. The coordinates for implantation of the DBS electrode to mPFC were AP +3.2 mm, L +0.6 mm, and DV 3.5 mm from Bregma. The animals were transferred to their home cages and kept under observation until awakened. They received 0.2 mg/kg s.c. meloxicam (Boehringer Ingelheim, Germany) for three days, and ampicillin (Ampicillin TZF, Polfa, Warsaw, Poland) was applied to the wound.

#### 2.5.2 Stimulation Procedure

After a 2-week postoperative recovery period, animals received stimulation (amplitude 250 μA, frequency 130 Hz; pulse width 90 μs) using a custom-made multi-channel stimulator (Plastics One Inc., Roanoke, VA, USA). Two 2-hour DBS sessions were conducted: one on the previous evening and one in the morning, 20 minutes before the sucrose consumption test and each of the three behavioural tests (Fig. **1**). The schedule was the same as used in previous studies [10, 13]. Animals in the SHAM group were treated in the same way, but the stimulator was switched off.

### 2.6 Behavioural Tests

#### 2.6.1 Elevated Plus Maze (EPM)

The animals were tested in two non-transparent boxes, which consisted of two open (50 × 11 cm, L × W) and two closed (50 × 11 × 40 cm) arms. The apparatus was elevated 50 cm above the floor and was illuminated by 25 W bulbs beneath each of the open arms. The number of entries into, and the time spent in the open and closed arms, were manually recorded (precision ± 0.1 s) during a 5-minute test.

#### 2.6.2 Novel Object Recognition (NOR)

The animals were tested in two non-transparent open fields (100 cm in diameter, 35 cm high, with the floor divided into painted 16 cm squares). Following two 10-minute adaptation sessions (days 1 and 2), the animals were allowed to explore freely two identical objects (day 3, white wooden cylinders, 7 cm in diameter, 11 cm high). The final criterion is 15 seconds of exploration of both objects. In a retention trial conducted 1 hour later (T2), one of the previously presented objects was replaced with a novel object (a black wooden prism, 5 cm wide and 14 cm high). The duration of exploration of each object was manually recorded during a 5-minute test. An NOR index was calculated according to the Akkerman [31] with the following formula: ((time of novel object exploration) - (time of familiar object exploration)/(total time of novel object exploration) + (total time of familiar object exploration)). The number of line crossings was measured as locomotor activity.

#### 2.6.3 Social Interaction (SI)

The animals were tested in two non-transparent open fields (100 cm in diameter, 35 cm high). After the first day of adaptation (10 minutes), on the next day, two unfamiliar rats of the same sex and matched for body weight (±10 grams) were put into the opposite ends of the arena. Time spent on active social behaviours, *i.e*., following, oronasal, body, and anogenital sniffing, grooming, mounting, and rolling, was recorded over 5 minutes [26, 32].

### 2.7 Electrophysiology

#### 2.7.1 Brain Slice Preparation for Electrophysiological Recordings

Rats were anesthetized with isoflurane (Aerrane, Baxter, UK). Their brains were quickly removed and immersed in an ice-cold artificial cerebrospinal fluid (ACSF) bubbled with a mixture of 95% O_2_ and 5% CO_2_. Two or three frontal cortex slices obtained from each animal were cut using a vibrating microtome (Leica VT1000) and subsequently stored submerged in ACSF at 30 ± 0.5°C.

#### 2.7.2 Field Potential Recording and LTP Induction

A concentric Pt-Ir stimulating electrode (FHC, Bowdoin, ME, USA) was placed in layer V of the medial prefrontal cortex. Stimuli (duration: 0.2 ms) were applied at 0.033 Hz using a constant-current stimulus isolation unit (WPI). Field potentials (FPs) were recorded using a glass micropipette filled with ACSF (1-2 MΩ), which was placed in an overlying site in cortical layer II/III. FPs were amplified (Axoprobe 1A, Axon Instruments, Foster City, CA, USA), A/D converted at 10 kHz, and stored using Micro1401 interface and Signal 2 software (Cambridge Electronic Design, Milton, Cambridge, UK).

For each slice, at the beginning of the experiment, a stimulus-response curve of FPs was generated. To obtain the curve, stimulation intensity was gradually increased stepwise (21 steps; 0-100 μA). One response was recorded at each stimulation intensity. The stimulus-response curves were fitted using the Boltzmann equation: V_i_ = V_max_/(1 + exp (u - u_h_)/-S), where V_max_ is the maximum FP amplitude; u is the stimulation intensity; u_h_ is the stimulation intensity that evokes an FP of half-maximum amplitude; S is the factor proportional to the slope of the curve.

LTP was induced by theta burst stimulation (TBS) at a stimulation intensity adjusted to evoke a response of 30% of the maximum amplitude of FPs. TBS consisted of ten trains of stimuli at 5 Hz, each composed of five pulses at 100 Hz, repeated 5 times every 15 seconds. During TBS, the pulse duration was increased to 0.3 ms. The amount of LTP was assessed in terms of an average increase in the amplitude of responses recorded between 45 and 60 minutes after the last burst relative to baseline.

### 2.8 Histological Verification of the DBS Electrode Implantation and Verification of the IBO Lesion in vHPC

After completion of the experiments, animals were anesthetized with isoflurane and sacrificed by decapitation; their brains were removed, and the correct placement of electrodes was verified in frozen coronal sections of mPFC (Figs. **2A** and **B**). Animals, in which the electrode tips were found to be outside the mPFC (up to 2%), were excluded from the data analysis.

The brains were collected after the lesion for verification of IBO-induced cell loss in vHPC. Brains were fixed in a 4% paraformaldehyde solution and then transferred to a 20% sucrose solution in PBS. The brains were frozen and sectioned at 30 μm on a cryostat (Leica CM3050S), around the injection site (Bregma -5.3/-5.6) and stained with 0.1% cresyl violet. Stained cells were counted stereologically in the bilateral CA1 vHPC region using a light microscope (Leica, Denmark) equipped with a projection camera (Basler Vision Technologies, Germany) and a stepper xyz stage (PRIOR ProScan) controlled by New CAST software (Visiopharm, Denmark). The number of stained cells was calculated at 100× magnification with immersion oil (Leica, Denmark) using a random meander sampling method. The area of the counting frame was 25000 μm 2 and covered 40% of the screen area. The meander sampling was applied to approximately 3% of the designated regions of interest, resulting in the counting of at least 100 cells from each sham animal. Neurons without a visible nucleus were not counted.

### 2.9 Drugs

Venlafaxine (VEN) (Carbosynth Ltd, Compton, Berkshire, UK) was dissolved in sterile 0.9% NaCl, which was used as a vehicle (SAL). The dose of VEN was the same as in previous studies [11, 12, 17, 18]. Ibotenic acid (Sigma-Aldrich Merck, cat no. I2765) was dissolved in sterile 0.9% NaCl, which was used for vehicle administration (SAL). The dose was based on previous studies [27-29, 33, 34] and our pilot, unpublished data.

### 2.10 Statistical Analysis

The data were checked for normality with the Kolmogorov-Smirnov test. The behavioural data were analyzed using analysis of variance (ANOVA) with three between-subject factors: group (CON *vs.* STR), lesion (SHAM *vs.* IBO), and treatment (SAL *vs.* VEN in Experiment 1, and SHAM *vs.* DBS in Experiment 2), and sucrose intakes as a within-subject factor. The electrophysiological data were analyzed using analysis of variance (ANOVA) with three between-subject factors: group (CON *vs.* STR), lesion (SHAM *vs.* IBO), and treatment (SAL *vs.* VEN in Experiment 1 or SHAM *vs.* DBS in Experiment 2). When significant interactions were found, a post hoc comparison was performed using Tukey’s test. The statistical analyses were performed using StatSoft, Inc.’s STATISTICA v. 10. The alpha value was set at the *p <* 0.05 level. All data are presented as means ± SEM.

## 3 RESULTS

### 3.1 Experiment 1

#### 3.1.1 Effect of VEN on the Reduction of Sucrose Consumption Caused by CMS Procedure and IBO Lesions in vHPC

As shown in Fig. (**3**), sucrose intakes were reduced by approximately 50% during the first two weeks of stress (weeks 6-8) and they remained at similar level throughout the experiment in SAL-treated animals (Group effect: (F(1,56) = 78,444, *p <* 0.001; Group x Weeks interaction (F(8,112) = 3,472, *p*=0.001). Chronic administration of VEN reversed the deficit in sucrose intakes, resulting in significant Treatment effect (F(1.56) = 3.974, *p*=0.049) and Treatment x Weeks interaction (F(7,112) = 4,053, *p <* 0.001). The IBO lesion in vHPC significantly decreased sucrose intakes (Group effect: F(1.28) = 5.911, *p =* 0.022). This effect was only partially attenuated by VEN (Treatment effect (F(1.28) = 8.565, *p =* 0.07). In consequence, at the end of the treatment period (Week 8), the intakes in stressed lesioned animals treated with VEN remained significantly lower than those in lesioned control groups (*p <* 0.001).

#### 3.1.2 Effect of VEN on Memory and Social Deficits Caused by Chronic Stress and IBO Lesion in vHPC

The results of the NOR and SI tests are shown in Fig. (**4**). The CMS procedure reduced exploration of novel objects and social interactions with an unfamiliar partner. Both of these effects were reversed by VEN, resulting in a significant Group (NOR: F(1.26) = 4.342, *p <* 0.05, SI: F(1.29) = 10.347; *p <* 0.001) and Treatment (NOR: F(1.28) = 8.659, *p <* 0.001, SI: F(1.29) = 11.505; *p <* 0.01) effects. IBO lesion in vHPC caused similar effects, resulting in a significant Group (NOR: F(1.28) = 10.504; *p <* 0.01; SI: F(1.29) = 3.027; *p =* 0.05) effect. As expected, VEN was inactive against the IBO-induced deficit in the NOR test (Treatment effect: F(1.28) = 2.522; NS) but it improved the lesion-induced reduction in social responses (Treatment effect: F(1.30) = 7.789; *p <* 0.01).

#### 3.1.3 Effect of VEN on Enhanced Anxiety Caused by Chronic Stress and IBO Lesion in vHPC

The results of the EPM test are shown in Fig. (**4**). As in other tests, the CMS procedure reduced the open arm entries, and this effect was reversed by VEN. In consequence, ANOVA revealed a significant Group (F(1.29) = 4.345; *p <* 0.05) and Treatment (F(1.27) = 29.602; *p <* 0.001) effects. IBO lesion in vHPC did not increase the anxiety-like responses of stressed animals (Group effect: F(1.29) = 0.369; NS), and VEN caused a clear anxiolytic-like effect in lesioned animals (Treatment effect: F(1.29) = 13.285; *p <* 0.01).

### 3.2 Experiment 2

#### 3.2.1 Effect of DBS on Reduction of Sucrose Consumption Caused by CMS Procedure and IBO Lesions in vHPC

As in the previous experiment, sucrose intakes were reduced by at least 50% during the first two weeks of stress (week 6-8) and they remained low throughout the experiment in SHAM animals (Group effect: (F(1.14) = 6.52, *p <* 0.05; Group x Weeks interaction (F(5.70) = 1.95, *p =* 0.098, NS). (Fig. **5**) DBS fully reversed this effect, resulting in significant Treatment effect (F(1.14) = 6.71, *p <* 0.01) and Treatment x Weeks interaction (F(5.70) = 4.83, *p <* 0.001). IBO lesion in vHPC also significantly decreased sucrose intakes (Group effect: F(1.27) = 19.767, *p <* 0.001). DBS reversed this effect, resulting in significant Treatment (F(1.13) = 7.72, *p <* 0.01) effect and Treatment x Weeks (F(5.65) = 5.68, *p <* 0.001) interaction.

#### 3.2.2 Effect of DBS on Memory and Social Deficits Caused by Chronic Stress and IBO Lesion in vHPC

The results of the NOR and SI tests are shown in Fig. (**6**). The CMS procedure reduced exploration of novel object and social interactions with unfamiliar partner and both these effects were reversed by DBS, resulting in a significant Group (NOR: F(1.26) = 4.344; *p <* 0.05, SI: F(1.26) = 7.389, *p <* 0.05) and Treatment (NOR: F(1.26) = 20.229, *p <* 0.001, SI: F(1.24) = 24.466; *p <* 0.001) effects. IBO lesion in vHPC caused similar effects, causing a significant Group (NOR: F(1.25) = 8.829; *p <* 0.01; SI: F(1.27) = 0.011; NS) effect. DBS was active against the IBO lesion-induced deficit in NOR (Treatment effect: F(1.26) = 20.229; *p <* 0.001) and SI (Treatment effect: F(1.24) = 24.466; *p <* 0.001) tests.

#### 3.2.3 Effect of DBS on Enhanced Anxiety Caused by Chronic Stress and IBO Lesion in vHPC

The results of the EPM test are shown in Fig. (**6**). As in other tests, CMS procedure reduced the open arm entries, and this effect was reversed by DBS leading to a significant Group (F(1.28) = 4.089; *p <* 0.05) and Treatment (F(1.28) = 7.112; *p <* 0.01) effects. IBO lesion in vHPC did not increase the anxiety-like responses of stressed animals (Group effect: F(1.28) = 0.001; NS), and DBS caused an anxiolytic-like effect in lesioned animals (Treatment effect: F(1.28) = 7.112; *p <* 0.01).

### 3.3 Experiment 3

#### 3.3.1 CMS Procedure Impairs mPFC Synaptic Plasticity, and IBO Lesion in vHPC Potentiates effects of CMS Procedure by Decreasing mPFC Basal Synaptic Transmission

ANOVA of the maximum calculated amplitude of FPs in mPFC slices revealed a significant Stress (F(1.134) = 27.03, *p <* 0.001) effect and Stress x Lesion (F(1.134) = 5.148, *p =* 0.025 interaction. The maximum FP amplitude in control animals reached 1.505 ± 0.073 mV; in slices obtained from rats subjected to CMS, the maximum FP amplitude was slightly lower (1.408 ± 0.076 mV), but this difference was not statistically significant (*p =* 0.99). In contrast, maximum FP amplitude in slices from CON IBO rats reached 1.629 ± 0.058 mV, in slices from CMS IBO rats, maximum FP amplitude was markedly decreased to 1.058 ± 0.066 mV (*p <* 0.001; Fig. **7A**).

ANOVA of LTP amplitude confirmed a statistically significant effect of Stress (F(1.108) = 19.64, *p <* 0.001). LTP was impaired by CMS irrespective of whether or not rats received IBO infusion into vHPC (Fig. **7B**). While the maximum amplitude of LTP in control animals reached 129.3 ± 4.52%, in CMS rat slices, the amplitude of LTP was decreased to 109.8 ± 3.94% (*p =* 0.033). Similarly, while the amplitude of LTP in slices from unstressed vHPC lesioned animals reached 142.5 ± 4.98%, in slices obtained from lesioned CMS rats, the amplitude of LTP was decreased to 107.7 ± 5.35% (*p <* 0.001). Both maximum FP and LTP amplitude were higher, but not statistically significant, in the mPFC of unstressed lesioned animals (Figs. **7A**, **B**).

#### 3.3.2 VEN Prevents the Reduction of FPs and LTP Loss Caused by Stress and IBO Lesion in vHPC

Treatment with VEN resulted in a recovery of the FP amplitude in the mPFC of stressed rats with vHPC lesion. The maximum FP amplitude in stressed lesioned animals that received saline was significantly lower than in their respective controls (1.058 ± 0.065 mV *vs.* 1.629 ± 0.058 mV, respectively, *p <* 0.001). However, maximum FP amplitude in stressed lesioned animals treated with VEN was not significantly different from that in their respective controls (1.388 ± 0.074 mV *vs.* 1.57 ± 0.09 mV, respectively, *p* = 0.457) (Figs. **8A**, **B**). Regarding the amplitude of LTP, ANOVA revealed a significant Treatment (F(1.108) = 5.018; *p =* 0.027) effect, and Treatment x Groups (F(1.108) = 22.62, *p <* 0.001) interaction (Figs. **8C**, **D**). While LTP was impaired by CMS in both IBO-lesioned and non-lesioned animals, in mPFC slices obtained from VEN-treated, stressed rats, the amplitude of LTP was similar to that in unstressed rats (123.3 ± 4.20% *vs.* 128.6 ± 5.24%, respectively, *p =* 0.99). LTP amplitude was significantly higher in CMS VEN-treated rat slices compared to SAL (128.6 ± 5.24% *vs.* 109.8 ± 3.94%, respectively, *p =* 0.022).

In slices prepared from VEN-treated, stressed rats with vHPC lesions, the amplitude of LTP was similar to that in slices prepared from non-stressed rats (131.0 ± 4.75% *vs.* 130.7 ± 4.75%, respectively, *p <* 0.99) and significantly higher than in slices obtained from SAL stressed rats (107.7 ± 5.35% *vs.* 130.7 ± 4.75%, respectively, *p =* 0.009).

#### 3.3.3 DBS Restores FPs Amplitude but Dampens the Induction of LTP in the mPFC of Stressed Animals with vHPC Lesions

Three-way ANOVA analysis of maximum FP amplitude revealed a significant DBS (F(1.115) = 5.526, *p =* 0.020), Lesion (F(1.115) = 7.848, *p =* 0.006) and Stress (F(1.115) = 10.30, *p =* 0.017) effects, as well as STR x DBS (F(1.115) = 4.213, *p =* 0.042), STR x IBO (F(1.115) = 11.06, *p =* 0.001) and STR x DBS x IBO (F(1.115) = 16.68, *p <* 0.001) interactions. While maximum FP amplitude in DBS sham control animals reached 1.493 ± 0.113 mV, in slices obtained from rats subjected to CMS DBS sham, it was lower (1.371 ± 0.083 mV). This difference was not statistically significant (*p* > 0.05). In contrast, while maximum FP amplitude in slices from non-stressed lesioned animals reached 1.706 ± 0.053 mV (mean ± SEM), in slices obtained from lesioned rats subjected to CMS, maximum FP amplitude was markedly lower (1.044 ± 0.049 mV; *p <* 0.001) (Fig. **9A** and **B**). This result confirms that pairing a vHPC lesion with CMS results in a decrease of basal synaptic transmission in the mPFC. DBS resulted in a recovery of FP amplitude in the mPFC of stressed rats with a lesion. Maximum FP amplitude was significantly higher in DBS-stressed rats than in the sham group (1.582 ± 0.119 mV *vs.* 1.044 ± 0.049 mV, respectively, *p <* 0.001). The maximum FP amplitude in stressed lesioned animals receiving DBS was not significantly different from that of their control (1.582 ± 0.119 mV *vs.* 1.463 ± 0.056 mV, respectively, *p =* 0.956). These findings suggest that DBS of the mPFC reversed the detrimental effect of CMS on mPFC basal synaptic transmission in rats with IBO-induced vHPC lesion.

The amplitude of LTP was also affected by DBS showing a significant Group (F(1.99) = 4.480, *p =* 0.037), Lesion (F(1.99) = 14.32, *p =* 0.0003), Stress (F(1.99) = 31.44, *p <* 0.001) effects, as well as DBS x IBO interaction (F(1.99) = 15.12, *p <* 0.001) (Figs. **9C** and **D**). In the mPFC of DBS sham rats, LTP was impaired by CMS irrespective of whether or not rats received IBO infusion into vHPC. While the maximum amplitude of LTP in control animals reached 129.2 ± 4.73%, in slices from CMS rats, the amplitude of LTP was decreased to 110.0 ± 4.08% (*p <* 0.05). Similarly, while the amplitude of LTP in slices from control lesioned animals reached 141.1 ± 4.76%, in slices obtained from CMS lesioned rats, the amplitude of LTP was decreased to 108.7 ± 5.10% (*p <* 0.01).

In mPFC slices obtained from stressed, DBS-stimulated rats, the amplitude of LTP was not significantly different from that of non-stressed rats (121.9 ± 8.66% *vs.* 115.1 ± 3.91%, respectively, *p =* 0.98). In slices prepared from DBS-stimulated, stressed rats with vHIP lesions, FPs were reduced after delivering theta-burst stimulation (TBS) meant to induce LTP. While in slices prepared from non-stressed lesioned DBS rats, the amplitude of LTP reached 107.8 ± 3.72%, in slices from stressed lesioned DBS rats it was decreased to 83.10 ± 11.28% (*p =* 0.032), indicating that instead of inducing LTP, DBS resulted in long-term depression (LTD).

### 3.4 Histological Verification of the IBO Lesion in vHPC

 The analysis of stained cells in the CA1 region of vHPC showed a 16% and 26% reduction in the number of neurons after IBO microinjection 2 and 5 weeks, respectively, after the lesion (Table **1**), and an increase in glial cells of up to 49% 2 weeks after the lesion. These effects did not reach statistical significance, which was probably caused by unusually high SEMs.

The difference in the organization of hippocampal CA1 layers between control and ibotenic acid-treated animals is shown in Fig. (**2B**).

## 4 DISCUSSION

The study showed that the CMS procedure decreased sucrose consumption (a measure of anhedonia), increased anxiety, and caused cognitive and social deficits. These findings are consistent with earlier studies with this procedure [14, 15, 22]. Infusions of IBO into vHPC produced comparable effects in stressed animals, except for anxiety-like responses, which were not affected by the lesion. Intra-HPC infusions of IBO were shown to cause degeneration of all local nerve cell bodies around the injection cannula. In contrast, axons of passage and nerve terminals of extrinsic origin seemed not to be affected. This was accompanied by some behavioural changes, such as enhanced anxiety, cognitive deficits, and altered responsiveness to sudden stressors, particularly those related to novelty or exploration-oriented behaviors [28, 29, 35].

In this study, bilateral injection of IBO caused a reduction in the number of hippocampal cells in the CA1 region, as well as enhanced anxiety and deficits in cognitive functions and social responses. Analysis of neuroanatomical changes after an excitotoxic lesion induced by microinjection of IBO showed extended cell loss around the place of injection, *e.g*., including the vHPC, lateral hypothalamus, and mPFC, and other connected structures [28, 36-38]. In this study, IBO infusion caused a loss of cells in the CA1 region and an increase in glial cells in response to injury. These findings confirm the validity of using ibotenic acid to induce a discrete structural lesion. Morphological deficits and the induction of behavioural changes confirm that the lesion can be used to model a situation in which the vHPC to mPFC pathway is impaired. The importance of intact vHPC to mPFC transmission in the mechanism(s) of antidepressant action was first suggested by a study showing that optogenetic or chemogenetic activation of the vHPC-mPFC pathway elicited antidepressant-like effects in the forced swim test [39]. Conversely, either functional disconnection or optogenetic inhibition of this pathway blocked the sustained antidepressant-like effect of ketamine in the forced swim test. A similar effect of vHPC electrical stimulation on behaviour in the forced swim test was also reported in WKY rats [40]. Finally, it was shown that long-term potentiation (LTP) at mPFC synapses following tetanic stimulation of vHPC in anesthetized rats was increased by chronic, but not acute, treatment with fluvoxamine [41], suggesting that this may also apply to the mechanism of action of conventional antidepressants.

Chronic administration of VEN was effective against all effects in non-lesioned stressed animals. However, in IBO-lesioned animals, VEN was inactive in reversing cognitive and social deficits in the NOR and SI tests and only partially normalized the hedonic behaviour in the sucrose test. Thus, in situations where the vHPC is inactivated as a result of the IBO lesion in Wistar rats, or its functions are somehow impaired in WKY rats, the main point of action for VEN remains the mPFC, and this appears to be insufficient to achieve full recovery of depressive-like symptoms. This possibility is further supported by our previous findings, which show that the antidepressant non-responsiveness of the WKY rat to chronic administration of VEN can be completely overcome by concurrent optogenetic stimulation of afferent terminals in the mPFC [22]. On the other hand, the ability of VEN to cause anxiolytic-like effects in IBO-lesioned animals observed in this study suggests that the mechanism of this action is localized beyond the vHPC-mPFC pathway. One candidate may be the amygdala complex, which receives strong projections from the mPFC, which in turn projects back to the amygdala, thus making direct reciprocal connections between the two structures. Activation of the mPFC-amygdala transmission causes anxiolytic-like effects both in naive animals and in animals exposed to various anxiety- and depression-related procedures [42-45]. Importantly, the increased anxiety induced by some of these procedures was also attenuated by VEN, and this effect was accompanied by changes in gene expression and increased neurogenesis in mPFC and amygdala [45]. Thus, it can be speculated that the anxiety-like effect of VEN observed in our vHPC-lesioned animals was related to its action within the mPFC, with no restoration of function in the vHPC to mPFC pathway. Such an effect could represent direct drug actions within the mPFC, bypassing HPC. It is possible that the improvement in social behavior observed in this study by VEN also involves a similar mechanism. These hypotheses require further evaluation, and we are currently verifying them in our ongoing studies.

Contrary to the effect of VEN described above, all deficits caused by the CMS procedure and the IBO lesion in vHPC, *i.e*., decrease of sucrose intakes, reduction of open arm entries, decrease of novel object exploration and social interaction episodes, were fully reversed by single sessions of DBS in mPFC. Similar effects of this treatment on the CMS-induced behavioural effects were observed in our previous studies [13, 14, 17, 46] as well as those reported by others [10, 23, 47-49] and not only in depression models [50-53]. However, this study is not aware of any other reports showing the efficacy of DBS in the mPFC in animals with IBO lesion in vHPC. These results indicate that DBS is effective as an antidepressant treatment in both animals with a dysfunction of the vHPC-mPFC pathway caused by chronic stress and in situation where the exclusion of this pathway is permanent, *i.e*., in WKY rats and following IBO lesion of vHPC. This is consistent with the concept that WKY rats and Wistar rats with IBO lesion in vHPC represent a reliable animal model of TRD and provides further support for the hypothesis that antidepressant resistance may reflect an insufficiency of transmission in the vHPC to mPFC pathway.

The antidepressant effect of DBS in mPFC, in both antidepressant-responsive (Wistar) and antidepressant-resistant (WKY) rats, is dependent on increased glutamate outflow in mPFC, acting locally through AMPA receptors [14]. Although it is unknown how the stimulation generates this effect, it may be assumed that a similar mechanism may also underlie the action of DBS in IBO-lesioned rats. This possibility is supported by the fact that normalization of the IBO effects occurred just a few minutes after the DBS session. In contrast, VEN took several weeks to only partially normalize the behaviour of these animals. The same rapid antidepressant-like effects are caused by ketamine [54-56], the mechanism of which involves, among others, the GluA1 subunit of the AMPA receptor [57-59]. Moreover, expression of these receptors in PFC is decreased by CMS [60] and normalized by DBS [10].

While it has been documented that different forms of chronic stress modify morphology and functions of mPFC neurons [61-64], we have not observed any significant reduction of FPs in mPFC slices obtained from the CMS rats. Since short-latency FPs in the rat mPFC are a product of activity of monosynaptic glutamatergic connections acting mainly *via* AMPA receptors with a minor contribution of NMDA receptors [65], this result indicates a lack of CMS-induced modifications of excitatory synaptic transmission in activated connections. It should, however, be noted that we recorded FPs in layer II/III of mPFC that receive long-range connections from the basolateral amygdala, mediodorsal thalamus, and contralateral mPFC [66]. Electrical stimuli delivered to a site underlying the recording electrode activate these fibers that ascend through layer V, in addition to local, intracolumnar connections arising from layer V neurons [66]. It has been reported that CMS reduces the number of excitatory synapses in deep, but not superficial, layers of the mPFC [67, 68]. Moreover, chronic unpredictable stress resulted in a decrease in the frequency of miniature excitatory postsynaptic currents (mEPSCs) in layer V neurons of the mPFC [69].

The results of the present study indicate that in slices obtained from animals with IBO-induced hippocampal lesion that were subjected to CMS procedure, the amplitude of FPs recorded from layer II/III of the mPFC was strongly reduced. At variance with other long-range inputs, the projection from vHPC reaches mainly layers V and VI of the mPFC [66]. The vHPC to mPFC projection is composed of two parts that express specific patterns of synaptic connections with mPFC pyramidal neurons and different types of inhibitory interneurons [70]. In the mPFC, incoming synaptic signals are processed by a complex network involving both intra- and interlaminar connections [66]. It is tempting to speculate that the lack of vHPC-generated activity in the mPFC of rats with IBO-induced vHPC lesion, combined with a general effect of stress, results in profound modifications of the activity of local circuitry. Such a modified activity pattern would then produce a decrease in the efficacy of excitatory transmission that develops throughout the CMS procedure and also involves layer II/III of the mPFC. While this study has not investigated the underlying mechanism, it was noted that chronic stress-related impairment of excitatory transmission in the mPFC has been linked to an elevated corticosterone-induced reduction in BDNF release and TrkB signaling [61]. In line with previous studies, our data provide evidence that treatment with VEN ameliorated the detrimental effects of CMS procedure and IBO-induced lesion of vHPC [46]. Although the amplitude of FPs in slices from stressed rats with vHPC lesions that were treated with VEN was smaller than in the control group, this difference was not significant. Recent data demonstrated that the mechanism of VEN action may involve restoration of the balance of the excitatory to inhibitory transmission that is related to stimulation of the matrix metalloprotease-9 (MMP-9) [71].

Previous studies demonstrated that the mechanisms of synaptic plasticity in rodent mPFC can effectively be studied using slice preparations [72]. Importantly, the data of this study shows that although in mPFC slices originating from CMS-subjected control rats the amplitude of FPs remained unchanged, the induction of LTP was, nevertheless, impaired. A dissociation between the effects of CMS on basal synaptic transmission (a lack of change) and on the potential of the tested connections for synaptic plasticity (a decrease of LTP) indicates that underlying mechanisms are different and suggests that the latter is more vulnerable to factors associated with prolonged stress, most likely to chronically elevated corticosterone level [61]. It should be noted that both FP and LTP amplitudes were restored to control levels after treatment with VEN. In slices prepared from stressed rats with vHPC lesion that were subjected to DBS, we also observed restoration of FP amplitudes to control level, an effect superficially similar to that observed after treatment with VEN. However, the application of DBS produced strikingly different effects on LTP than in the case of VEN treatment. Firstly, DBS resulted in a marked reduction of LTP magnitude in slices prepared from unstressed rats with IBO-induced lesions. Secondly, in slices prepared from stressed rats with IBO-induced lesions, the same stimulation pattern that induced LTP in control preparations resulted in long-term depression. Such effects may be explained by the phenomenon of metaplasticity, a higher-order form of synaptic plasticity that does not change the efficacy of basal synaptic transmission but changes in the ability to induce subsequent modifications (LTP and LTD) [73]. It has been shown that metaplastic effects in mPFC may occur in relationship to behavioral changes [74]. It should be noted that DBS used in the present study involved stimulation of the mPFC *via* implanted electrodes by prolonged trains of pulses (2 hours of stimulation at 130 Hz gives 936.000 pulses). Remarkably, such a large number of stimuli did not modify basal synaptic transmission in nearby sites within the mPFC, as evidenced by a lack of change in FP amplitude in slices of the mPFC from rats subjected to DBS. An impairment of LTP and induction of LTD instead of LTP in the same slices is indicative of a shift of the sliding threshold for LTP induction in these slices towards higher values [75, 76]. In addition to local circuitry, prolonged stimulation of mPFC is likely to strongly activate the mPFC long-range outputs, including projections to striatum, amygdala, ventral tegmental area, as well as connections with other cortical areas [66]. Future work should address plausible modifications in all these projections by DBS.

## CONCLUSION

In conclusion, the CMS procedure causes several depression-relevant deficits, which can be effectively reversed by repeated administration of VEN and acute application of DBS in mPFC. IBO lesion in vHPC enhanced the CMS-induced anhedonia, cognitive and social impairments and caused neuronal loss in the CA1 region of vHPC, thereby attenuating glutamatergic transmission and long-term synaptic plasticity in mPFC. All these effects were fully normalized by DBS in mPFC but not by VEN, which only partially reversed the IBO lesion-induced effects. The weaker sensitivity of vHPC lesioned animals to the therapeutic action of VEN may provide further evidence that hippocampal insufficiency – and specifically, insufficiency of the vHPC to mPFC transmission – could be responsible for antidepressant non-response. In our opinion, these results have significant translational implication. Understanding the anatomical, cellular, and molecular substrates of antidepressant resistance is the basis for a better understanding of critical features of the central nervous system in TRD patients that are responsible for the lack of response to antidepressant treatment. Consequently, this should have obvious implications for the search for new strategies for treating TRD, indicating the need to search for targeted therapies, *i.e*., those whose mechanism of action involves a more selective activation of the neuronal networks we evaluate in this study.

## LIMITATIONS OF THE STUDY

This study has some potential limitations. Firstly, the studies were conducted only in male rats, while it is well-established that stress-related disorders occur depending on sex. Therefore, this study cannot be sure whether the findings can be extended to female rats. Furthermore, given that depression is diagnosed more frequently in women than in men, it would be valuable to repeat the study in female rats.

This study acknowledges that only one drug was used, and further studies investigating the effects of other antidepressants could provide valuable insights and enhance our understanding of the potential role of brain function in treating psychiatric disorders. Several antidepressants in CMS were tested and venlafaxine was chosen based on data obtained in previous experiments, its ability to normalise the anhedonic phenotype [17, 46], and its effectiveness in treating major depression.

Thirdly, the IBO lesion was studied in one brain region; however, the choice of structure was not accidental. This was based on the previous studies, which indicated that the ventral hippocampus is the main area for discriminating between antidepressant effects. Neither VEN nor vHPC–mPFC optogenetic stimulation alone was effective in reversing the effects of CMS; however, the combination of chronic VEN and repeated optogenetic stimulation restored normal behaviour. Conversely, optogenetic stimulation of dorsal hippocampus (dHPC-mPFC) did not affect sucrose intake [22].

## Figures and Tables

**Fig. (1):**
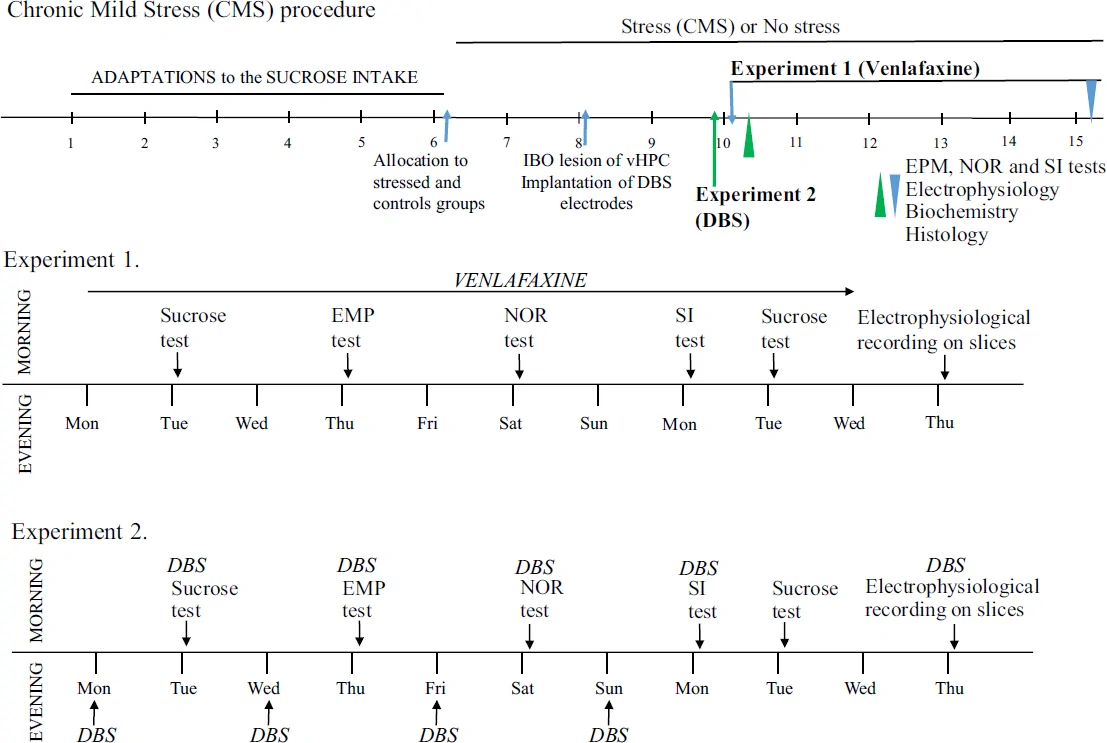
Diagrams showing the design of the CMS protocol and details of experiments 1 and 2.

**Fig. (2):**
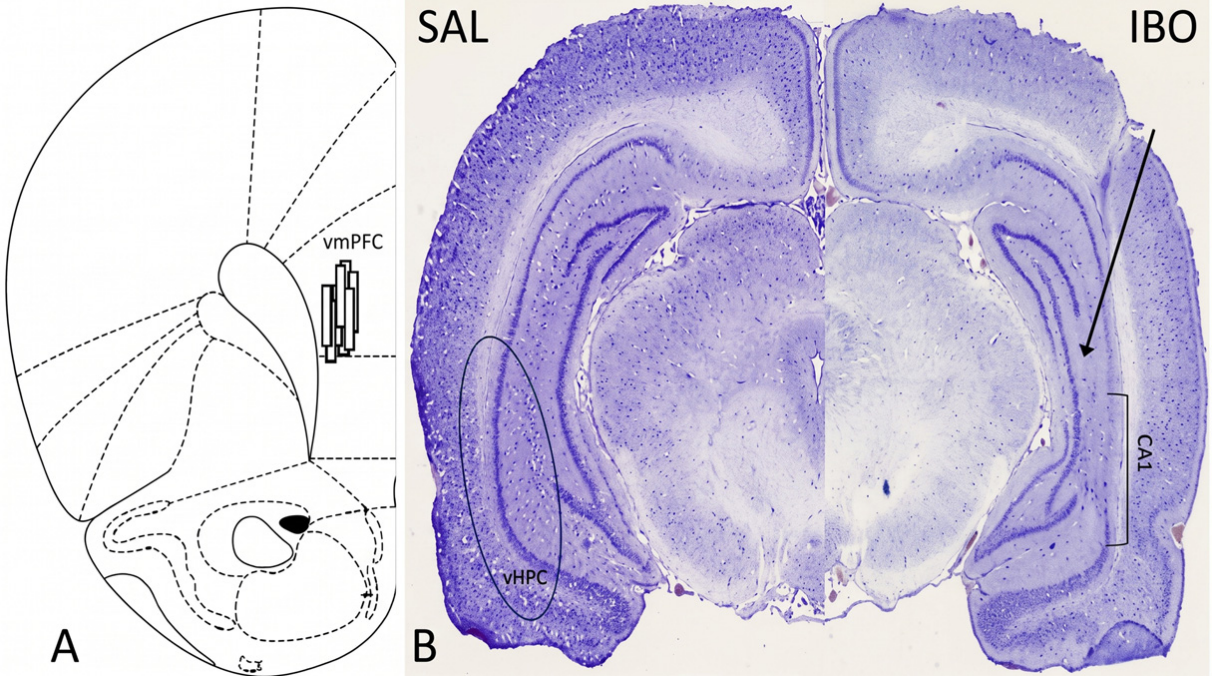
(**A**) Schematic illustration of the location of the DBS electrode tips in mPFC (3.2 mm coronal slice from Bregma; Paxinos, G. and Watson, C. (1995) The Rat Brain in Stereotaxic Coordinates. Academic Press, San Diego. (**B**) Representative photomicrographs showing comparison of SAL and IBO infusion in vHPC (coronal slice at -5.45 mm from Bregma; Paxinos, G. and Watson, C. (1995) The Rat Brain in Stereotaxic Coordinates. Academic Press, San Diego. The arrow points to the site of damage. Nissl staining revealed a disruption of hippocampal structure in the CA1 region after IBO lesion compared to the SHAM group.

**Fig. (3):**
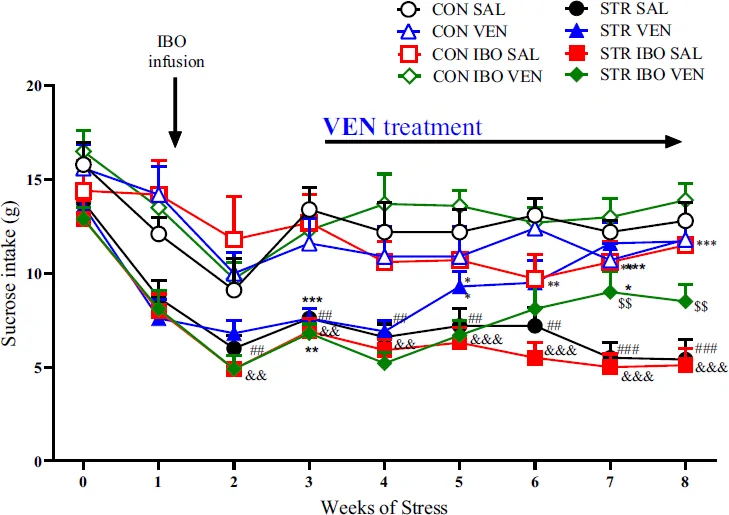
Effects of IBO lesion in vHPC and chronic venlafaxine (VEN, 10 mg/kg/day, IP) administration on the consumption of 1% sucrose solution in control (CON, open symbols) and stressed (STR, closed symbols) animals. Data are presented as means ± SEM. N = 8. ^##^-*p <* 0.01; ^###^-*p <* 0.001 relative to sucrose intakes at Week 0 for STR SAL group; **-*p <* 0.01; ***-*p <* 0.001; relative to sucrose intakes in STR SAL; ^&&^-*p <* 0.01; ^&&&^- *p <* 0.001 relative to sucrose intakes at Week 0 for STR IBO group; ^$$^-*p <* 0.01; relative to sucrose intakes in STR IBO SAL.

**Fig. (4):**
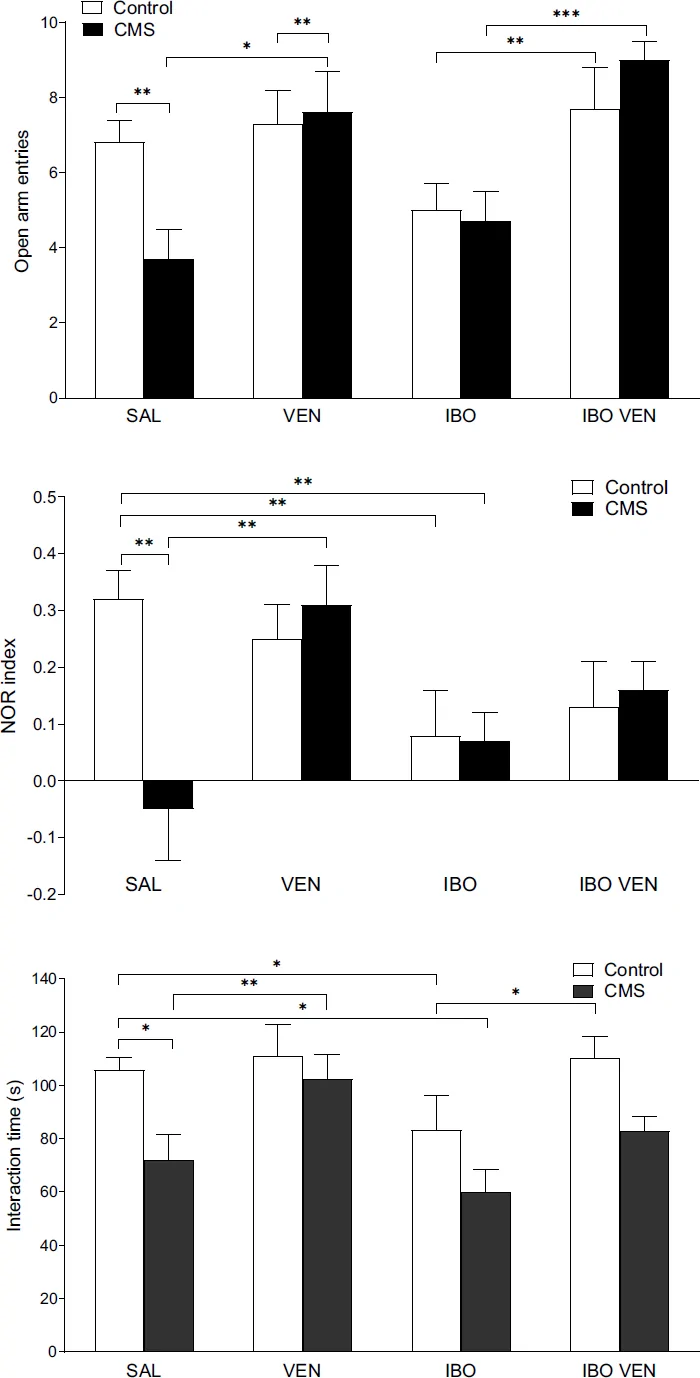
Effects of IBO lesion in vHPC and chronic venlafaxine (VEN, 10 mg/kg/day, IP) administration on the behaviour of control (open bars) and stressed (closed bars) animals in Elevated Plus Maze (EPM), Novel Object Recognition (NOR) and Social Interaction (SI) tests. Data are presented as means ± SEM. N = 7-8. *p < 0.05, **p < 0.01, ***p < 0.001.

**Fig. (5):**
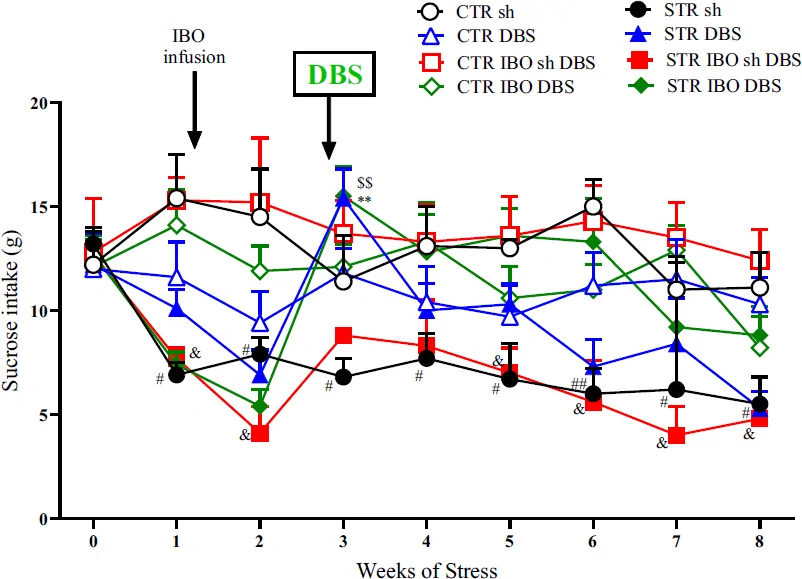
Effects of IBO lesion in vHPC and DBS in mPFC on the consumption of 1% sucrose solution in control (CON, open symbols) and stressed (STR, closed symbols) animals. Data are presented as means ± SEM. N = 8. ^##^-*p <* 0.01; ^###^-*p <* 0.001 relative to sucrose intakes at Week 0 for STR SAL group; **-*p <* 0.01; ^***^-*p <* 0.001; relative to sucrose intakes in STR SAL; ^&&^-*p <* 0.01; ^&&&^-*p <* 0.001 relative to sucrose intakes at Week 0 for STR IBO group; ^$$^-*p <* 0.01; relative to sucrose intakes in STR IBO SAL.

**Fig. (6):**
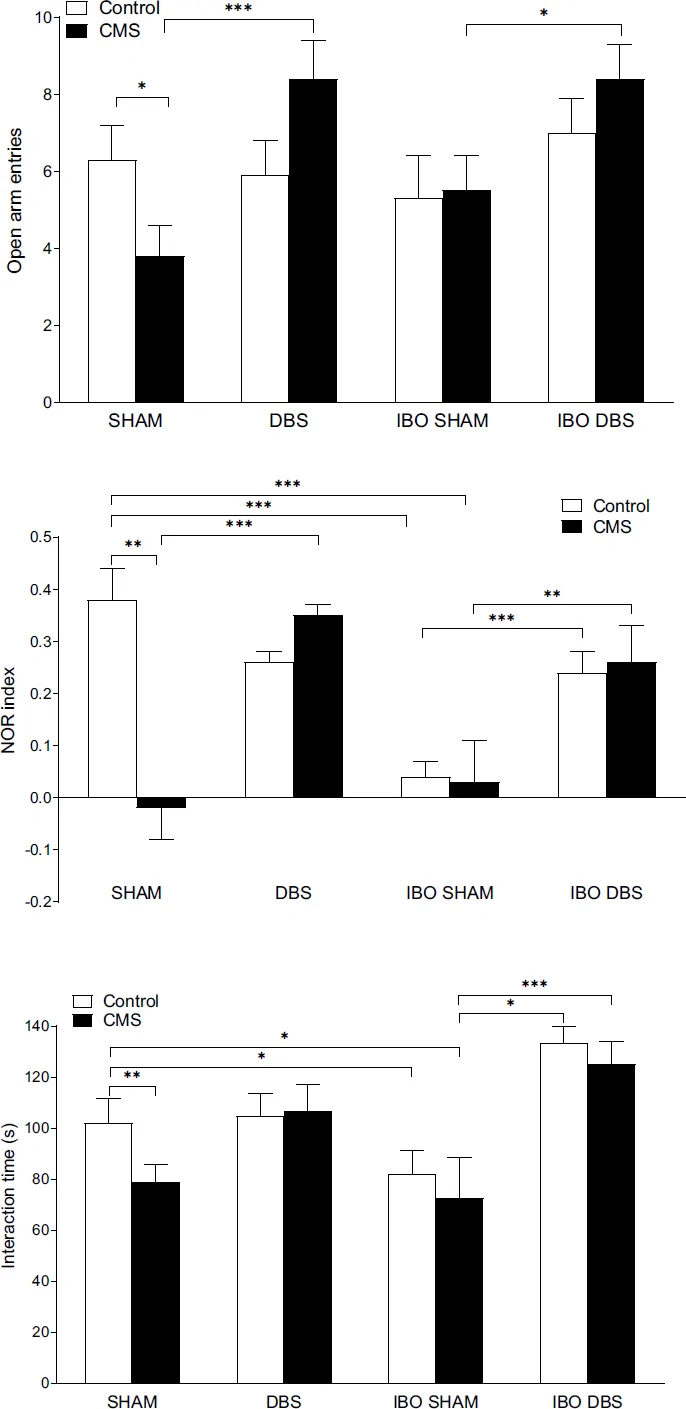
Effects of IBO lesion in vHPC and DBS in mPFC on the behaviour of control (open bars) and stressed (closed bars) animals in Elevated Plus Maze (EPM), Novel Object Recognition (NOR) and Social Interaction (SI) tests. Data are presented as means ± SEM. N = 7-8. *p < 0.05, **p < 0.01, ***p < 0.001.

**Fig. (7):**
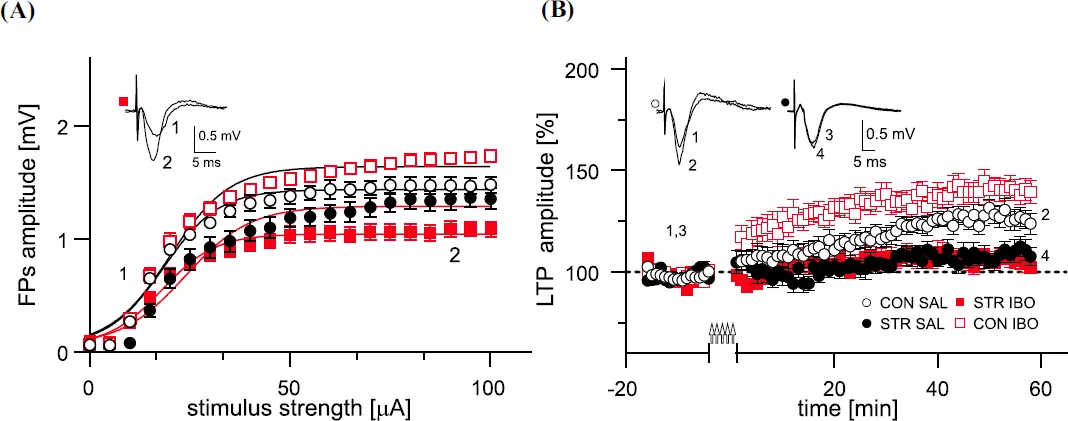
Effects of IBO lesion in vHPC on local field potentials (**A**) and LTP (**B**) recorded in the mPFC slices from control (open symbols, CON) and stressed (closed symbols, STR) animals. Figures present the relationship between stimulus intensity and mean FP amplitude and the time course of FP amplitude before and after LTP induction (arrows on B). Data are presented as means ± SEM. N = 4 in triplicate.

**Fig. (8):**
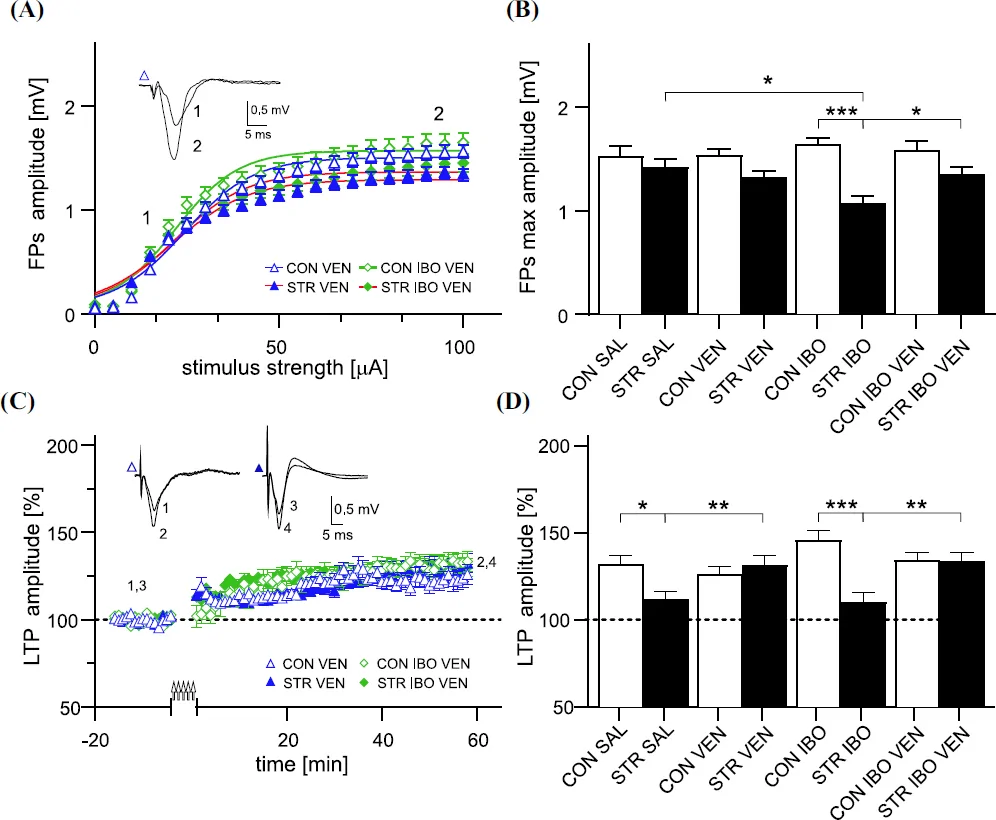
Effects of chronic venlafaxine (VEN, 10 mg/kg/day, IP) administration in animals with IBO lesion in vHPC on local FPs (**A**), Boltzmann max calculated amplitude (**B**), LTP (**C**), and LTP amplitude (**D**), recorded in mPFC slices from control (open symbols, CON) and stressed (closed symbols, STR) animals. Figures show the relationship between stimulus intensity and mean FPs amplitude and time course of FPs amplitude, before and after LTP induction (arrows on C). Data are presented as means ± SEM. N = 4 in triplicate. *p < 0.05, **p < 0.01, ***p < 0.001.

**Fig. (9):**
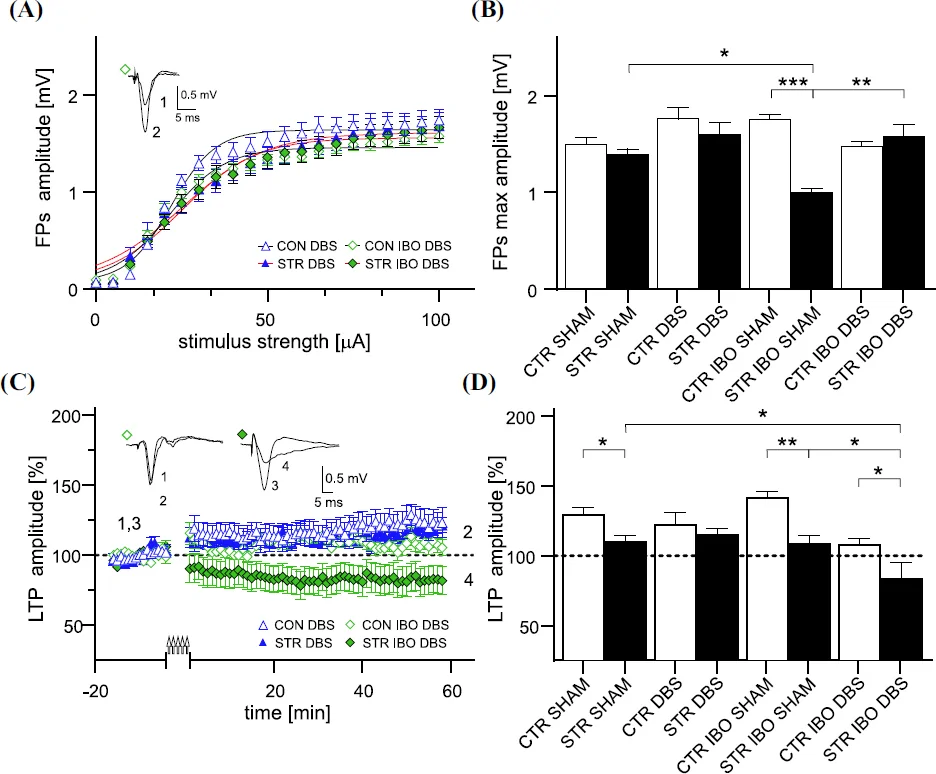
Effects of DBS in mPFC in animals with IBO lesion in vHPC on local fields potentials (**A**), Boltzmann max calculated amplitude (**B**), LTP (**C**), and LTP amplitude (**D**), recorded in mPFC slices from control (open symbols, CON) and stressed (closed symbols, STR) animals. Figures present the relationship between stimulus intensity and mean FP amplitude and the time course of FP amplitude before and after LTP induction (arrows on C). Data are presented as means ± SEM. N = 4 in triplicate. *p < 0.05, **p < 0.01, ***p < 0.001.

**Table 1:** The percentage and number of neurons and glial cells (%) in the CA1 region of vHPC after IBO lesion.

**-**	**Neurons (%) (nr)**	**Glial Cells (%) (nr)**	**Number of all Cells in the CA1 ROI** **(Number of Cells on m_3_/V CA1) (%)**
CONTROL	100 (148 ± 14)	100 (62 ± 9)	100 (251,17 ± 31,86)
IBO lesion 2 weeks	84 (123 ± 9)	151 (94 ± 17)	92 (231,82 ± 14,04)
IBO lesion 5 weeks	74 (110 ± 22)	102 (63 ± 13)	82 (206,06 ± 33,31)

## Data Availability

All data generated or analyzed during this study are included in this published article.

## LIST OF ABBREVIATIONS

CMS: Chronic Mild Stress
DBS: Deep Brain Stimulation
EPM: Elevated Plus Maze
FPs: Field Potentials
IBO: Ibotenic Acid
LTP: Long Term Potentiation
mPFC: Medial Prefrontal Cortex
NOR: Novel Object Recognition
SI: Social Interaction
TRD: Treatment-resistant Depression
VEN: Venlafaxine
vHPC: Ventral Hippocampus
WKY: Wistar-Kyoto Rats
